# Considerations for practical dose equivalent assessment of space radiation and exposure risk reduction in deep space

**DOI:** 10.1038/s41598-022-17079-1

**Published:** 2022-08-10

**Authors:** Masayuki Naito, Satoshi Kodaira

**Affiliations:** National Institute of Radiological Sciences, National Institutes for Quantum Science and Technology, Chiba, 263-8555 Japan

**Keywords:** Space physics, Risk factors

## Abstract

Shielding from space radiation, especially galactic cosmic rays (GCRs), is a significant safety challenge for future human activities in deep space. In this study, the shielding performances of potential materials [aluminum (Al), polyethylene (PE), and carbon fiber reinforced plastic (CFRP)] were investigated using Geant4 Monte Carlo simulation considering two types of biological scale parameters, the International Commission on Radiological Protection (ICRP) quality factor (QF_ICRP_) and the plausible biological effectiveness (RBE_γacute_), for GCRs. The effective dose equivalent was reduced by 50% for QF_ICRP_ and 38% for RBE_γacute_ when shielding using 20 g/cm^2^ of CFRP. A spacecraft made from CFRP will have a better radiation shielding performance than conventional Al-based spacecraft. The contribution of heavy ions for QF_ICRP_ based effective dose equivalent was larger by a factor of ~ 3 compared to that for RBE_γacute_ based effective dose equivalent. The shielding materials efficiently reduced the effective dose equivalent due to ions with QF_ICRP_ > 3.36 and RBE_γacute_ > 2.26. QF_ICRP_ and RBE_γacute_ have advantages and disadvantages in quantifying the dose equivalent of space radiation, and the establishment of a standard parameter specified for a mixed radiation environment occupied by protons and heavy ions is necessary for practical dose assessment in deep space.

## Introduction

An important factor restricting future human activities in deep space is radiation exposure from galactic cosmic rays (GCRs) and solar energetic particles (SEPs). Serious radiation damage poses a health hazard to space crews. Current and previous human space activities have been carried out in low-Earth orbit (LEO), where charged particles are partially shielded by geomagnetic fields. Future missions to the moon, the National Aeronautics and Space Administration’s Deep Space Gateway^1^, Mars, and deep space will face the challenge of high radiation risks because of the small magnetic fields and long mission terms. In addition, space travel for the public will be offered as a commercial product in the future, meaning that long-term space stays will not be exclusively for space crew.

Most GCR particles are protons (~ 87%)^2,3^. The high linear energy transfer (LET) and biological effects of the high charge and energy (HZE) particles from He to Fe nuclei significantly contribute to the radiation dose e.g., Refs.^4,5^. Measurements by the Mars Science Laboratory indicated a dose equivalent of ~ 660 mSv or more during an Earth-Mars round trip flight and 0.64 mSv/day during a stay on the Mars surface^6,7^. Considering the International Commission on Radiological Protection’s (ICRP) public and occupational exposure limits of 1 and 50 mSv/y, respectively^8^, reliable evaluation of radiation risks and effective radiation shielding is a significant challenge for safe and sustainable manned space development.

To evaluate the radiation risks from HZE particles, the dose equivalent, which is defined by the integral of the absorbed dose and radiation quality factor (QF), is employed^9^. QF is a relevant parameter to relative biological effect (RBE)^10,11^. ICRP has released the fluence conversion coefficients for the absorbed dose, mean QF, and dose equivalent to human tissues and organs and their effective average^9^. Biological effects, such as simple and complex exchanges, gene mutation, and neoplastic transformation, have been investigated in terms of estimating cancer risks e.g., Ref.^12–14^. The QF provided by ICRP (1991)^15^ is a conventional value of RBE_max_ under low-dose (< 0.2 Gy) and low-dose rate (~ 0.05 Gy/h) gamma-rays in model biological systems and it is described by a simple function of LET. A reduction in the effective dose equivalent is a good indicator for a reduction in radiation risk. However, the RBE_max_ produces large uncertainties under various experiments, mainly due to the low yields of the biological effects of the low dose and dose rate gamma-rays^5,16–18^. An approach obtaining RBE_γacute_ values with relatively small uncertainties is to obtain them under acute conditions of high dose (0.5–3 Gy) gamma-rays^5,17^. The acute gamma-ray model has obtained consistent RBEs by using the linear response to epidemiological data of atomic bomb survivors^19,20^. Meanwhile, Cucinotta et al.^21^ suggested that the RBE_γacute_ might be insufficient to assess risk as well as QF due to restricted information on the physics of incident ions; e.g., ${Z}^{*2}/{\beta }^{2}$ in (Eq. 3). After that, Cacao et al.^22^ obtained the RBE_γacute_ response to particle LET and charge number based on experimental datasets of ^16^O, ^20^Ne, ^28^Si, ^48^Ti, and ^56^Fe exposure.

Radiation shielding is a strategy used to reduce radiation exposure risks. Passive shielding is an approach to absorb relatively low energy particles and break up HZE particles into lighter particles in the shielding material, resulting in dose reduction. HZE particle exposure and dose reduction by shielding materials have been studied through calculations and measurements using ground accelerators e.g., Ref.^23–31^ and in space modules e.g., Refs.^6,32–39^. Several types of shielding materials, such as aluminum (Al), polyethylene (PE, (C_2_H_4_)_n_), and carbon fiber reinforced plastic (CFRP), have been studied. Al is commonly used in spacecraft construction, PE is known to be an effective shielding material, and CFRP is a potential structural material with a relatively high shielding efficiency for spacecraft owing to its high mechanical strength^40,41^.

In this study, we discuss the effective dose equivalents due to GCR exposure based on two types of biological scale parameters under passive shielding, which will be crucial for evaluating radiation-induced risks during upcoming long-term stays in space.

## Results

### GCR fluences behind shielding materials

**Figure 1:**
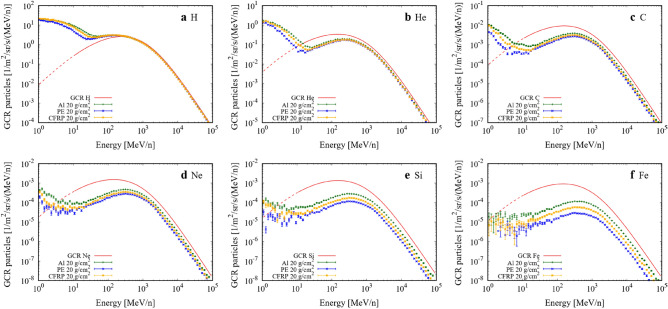
Energy spectra of GCR major elements in free space and with 20 g/cm^2^ shielding. (**a**) H, (**b**) He, (**c**) C, (**d**) Ne, (**e**) Si, and (**f**) Fe. Red line denotes GCR primary element in free space. Green, blue, and yellow denote GCR element with 20 g/cm^2^ shielding with Al, PE, and CFRP, respectively. Dotted energy regions (1–10 MeV/n) are not included in calculation primary sources because these low energy primary particles are stopped in a thin layer and do not contribute to the flux behind material.

Figure 1 shows the energy spectra of the major GCR elements in free space and behind the shielding materials: Al, PE, and CFRP. Error bars represent the statistical error of the calculation. Several space modules provide shielding of ~ 20 g/cm^2^ or more mass thickness on average^42,43^, therefore, we employed 20 g/cm^2^ as the typical thickness of spacecraft shielding. The proton flux was increased by shielding because the target and heavier projectile fragmentation reactions produced numerous secondary protons along the pathway of the primary particles in the materials. The number of heavy ions was decreased by the projectile fragmentation reactions, while some light ions at low energies (< 10 MeV/n) were increased by particle energy loss and secondary particle production owing to the target and heavier projectile fragmentations. Secondary particle production in the high energy region was highest in PE by projectile fragmentation, whereas that in the low energy region was highest in Al by target fragmentation. This is explained by the fragmentation cross section per unit mass^41^ and the particle production rate by fragmentation. PE has the largest cross section of the employed materials, followed by CFRP and Al. The fragmentation of heavier particles produces a larger number of particles e.g., Ref.^44^.

**Figure 2:**
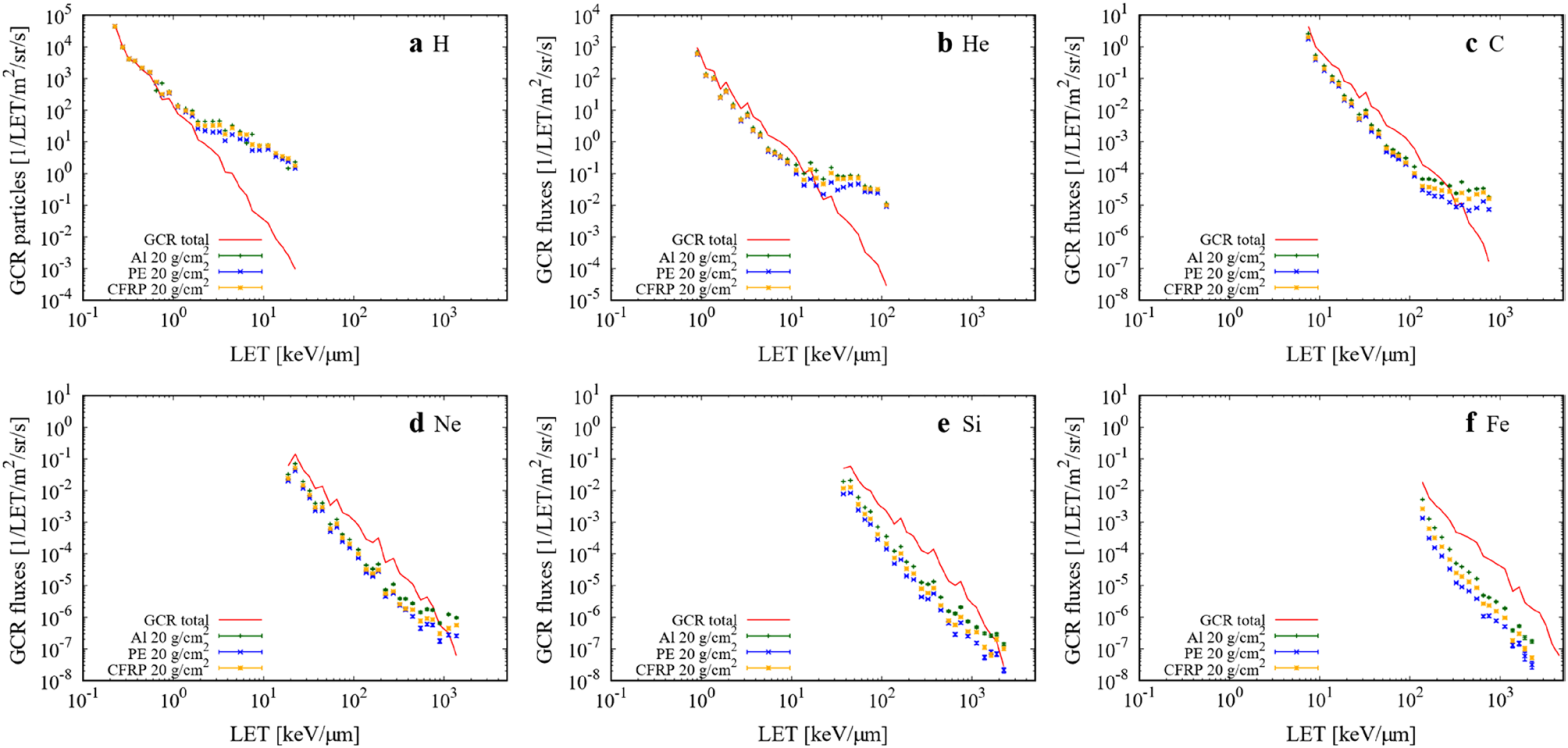
LET spectra of GCR major elements in ICRU four-element tissues in free space and with 20 g/cm^2^ shielding. (**a**) H, (**b**) He, (**c**) C, (**d**) Ne, (**e**) Si, and (**f**) Fe. Red line denotes GCR primary element in free space. Green, blue, and yellow denote GCR primary element with 20 g/cm^2^ shielding with Al, PE, and CFRP, respectively.

**Figure 3:**
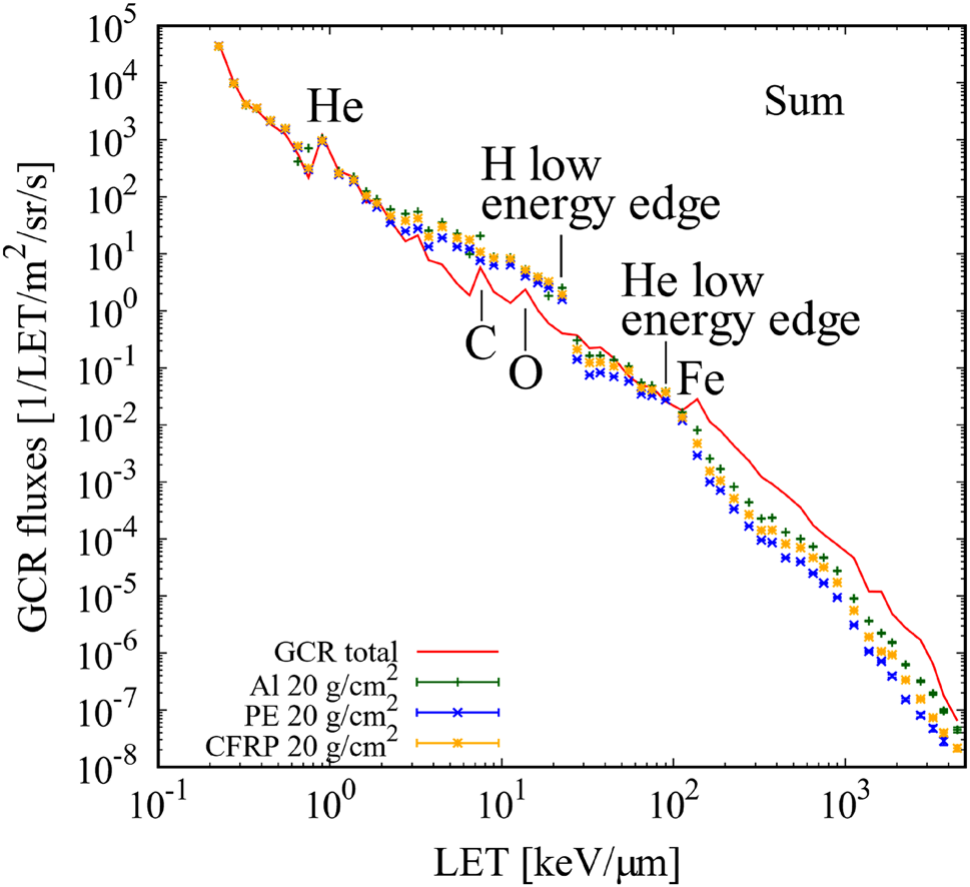
Summed LET spectra of Fig. 2a–f. Total GCR primary particles in free space (red line) and with 20 g/cm^2^ shielding with Al (green), PE (blue), and CFRP (yellow).

The energy spectra of the fluence were converted to LET-dependent fluence spectra in the ICRU four-element tissues^45^, as shown in Fig. 2. Summed LET spectra were obtained and are shown in Fig. 3. LET peaks for He, C, O, and Fe appeared at approximately 0.9, 7.5, 15, and 150 keV/µm, respectively, in free space. The mean QF_ICRP_ and RBE_γacute_ were obtained from these fluence spectra using Eqs. (2) and (3), respectively. Note that our results only include the contributions by the charged particles from protons to Fe ions; the contributions of neutrons, photons, pions, and muons were not considered. The shielding materials significantly reduced the flux of primary particles heavier than He owing to projectile fragmentation. The flux reduction rate increased with heavier nuclei; this was observed for Fe, which is one of the major contributors to the total dose. The shielding of high LET particles should contribute to the efficient reduction of biological effects. The increase in low energy H appeared as an enhancement at LET = 1–30 keV/µm. This was mainly because of the target fragmentations. Similar enhancements appeared in the heavier particles: He (30–100 keV/µm), C (500–1000 keV/µm), and Ne (1000–1500 keV/µm) (Fig. 2).

### Biological scale parameters

The absorbed dose rates and mean QF_ICRP_ and RBE_γacute_ of GCR elements in free space are summarized in Table 1. The total mean QF_ICRP_ and RBE_γacute_ values were obtained as follows:

**Table 1:** Absorbed dose rates and mean QF_ICRP_ and RBE_γacute_ of GCR elements in free space (with no shielding materials).

	${D}_{i}$ (mGy/y)	QF_ICRP_	RBE_γacute_
H	98.6	1.62	1.04
He	33.1	1.59	1.19
Li	0.26	1.89	1.39
Be	0.21	2.00	1.65
B	0.29	2.50	1.94
C	4.72	3.36	2.26
N	1.58	4.09	2.52
O	6.73	5.45	2.83
F	0.19	6.20	3.06
Ne	1.60	8.20	3.42
Na	0.45	9.90	3.61
Mg	2.39	10.41	3.77
Al	0.55	12.22	3.79
Si	2.55	13.65	4.06
P	0.15	14.89	4.14
S	0.64	16.18	4.06
Cl	0.16	17.45	4.25
Ar	0.32	18.61	4.13
K	0.26	19.59	4.29
Ca	0.64	20.06	4.17
Sc	0.14	20.62	4.14
Ti	0.52	20.52	4.04
V	0.29	19.99	3.90
Cr	0.61	19.39	3.76
Mn	0.65	18.90	3.66
Fe	4.46	18.60	3.55
Total mean	–	3.23	1.48

1 $$\langle QF (or RBE)\rangle =\frac{\sum Q{F}_{i} {D}_{i}}{\sum {D}_{i}},$$

where subscript $i$ is an element in the GCR particles (Z = 1–26). The total absorbed dose rate was 162 mGy/year and the QF_ICRP_ based effective dose equivalent (H_E(ICRP)_) was 523 mSv/year. The total mean QF_ICRP_ was larger than the total mean RBE_γacute_ by a factor of ~ 2.5. It should be noted that the effective dose equivalents based on QF_ICRP_ could not be overestimated by a factor of ~ 2.5. QF_ICRP_ is defined based on radiobiological data for conditions of low dose and low dose rate gamma-rays^15^. The difference due to the reference gamma-ray is compensated by the dose and dose rate effectiveness factor (DDREF)^15^. The value of the DDREF is a critical factor for obtaining the absolute value of the dose equivalent. The ICRP recommends a DDREF of 2^8,15^. However, the DDREF ranges from 2 to 5 depending on the targets and radiation quality e.g., Refs.^46–51^. The differences between QF_ICRP_ levels and RBE_γacute_ (Table 1) were consistent with the DDREF range.

**Figure 4:**
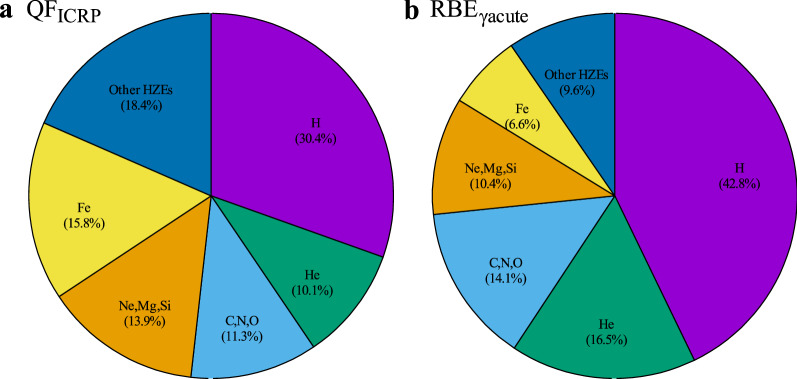
Contribution ratio of GCR particles to the effective dose equivalent. (**a**) QF_ICRP_ and (**b**) RBE_γacute_ in free space (with no shielding materials).

**Figure 5:**
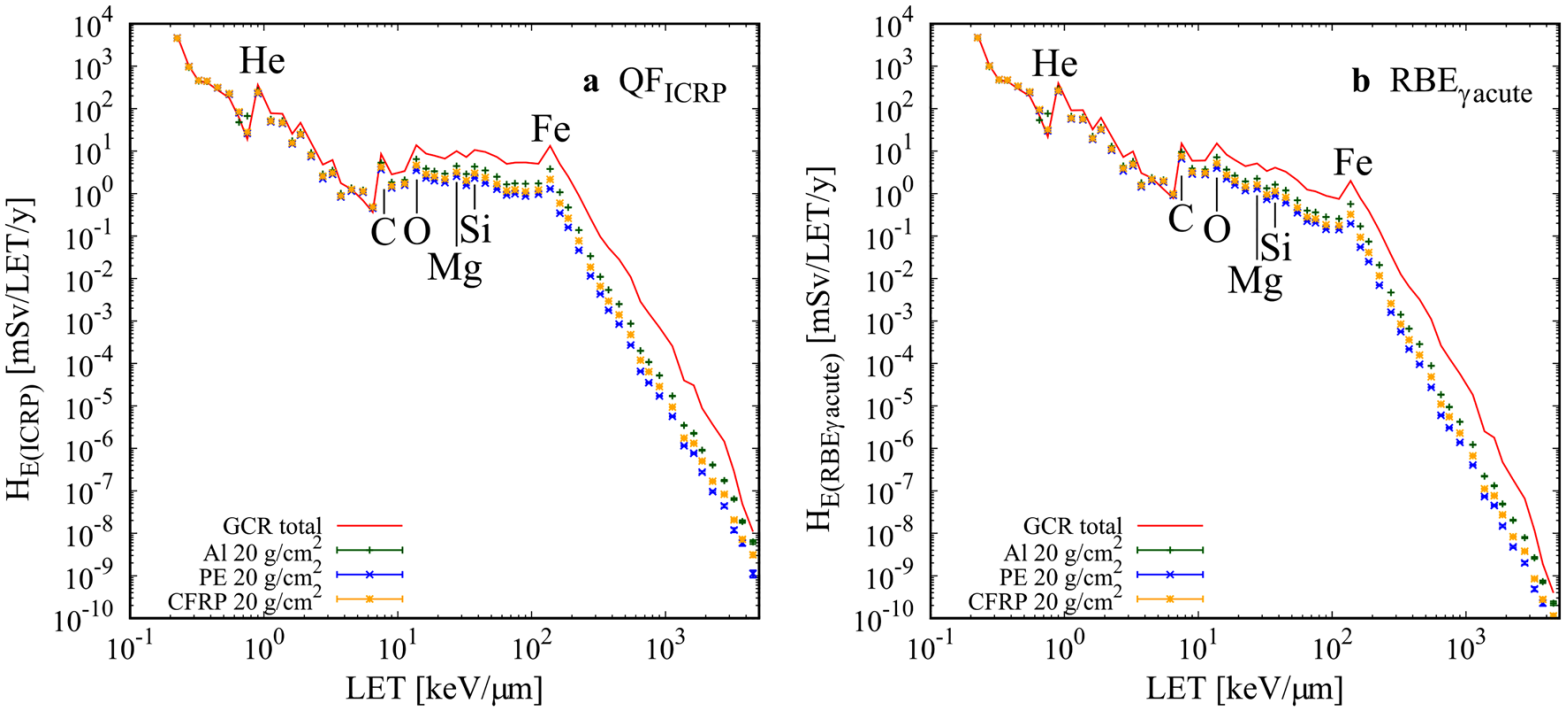
LET dependencies of the effective dose equivalents. (**a**) H_E(ICRP)_ and (**b**) H_E(RBEγacute)_. Red line denotes total GCR primary particles in the free space. Green, blue, and yellow denote total GCR primary particles with 20 g/cm^2^ shielding with Al, PE, and CFRP, respectively.

Figure 4 shows elemental contributions to the effective dose equivalents in free space. The contributions of H, He, C, N, and O to H_E(RBEγacute)_ were larger than their contributions to H_E(ICRP)_. The contribution of Fe to H_E(RBEγacute)_ was smaller than that to H_E(ICRP)_ by a factor of ~ 2.5. The LET dependences of effective dose equivalents in free space and with 20 g/cm^2^ Al, PE, and CFRP shielding are given in Fig. 5. The vertical axis of Fig. 5b, i.e., H_E(RBEγacute)_, does not consider the DDREF value. Therefore, direct comparisons between (a) and (b) cannot be made. The reduction rates of H_E(ICRP)_ and H_E(RBEγacute)_ were similar for the whole LET range. While the enhancement of the low energy and high LET particles generated in the shielding materials made a small variation in flux for LET < 100 keV/µm, the fragmentations from heavier ions than C contributed much to a dose reduction for LET > 8 keV/um. The mean QF_ICRP_ and RBE_γacute_ (Table 1) indicate that the shielding material is efficient at reducing the effective dose equivalent due to the high LET particles of QF_ICRP_ > 3.36 or RBE_γacute_ > 2.26. The dose reduction by CFRP was by a factor of ~ 2 at 10 keV/µm, ~ 5 at 100 keV/µm, and ~ 25 at 1000 keV/µm. The variations of relative absorbed dose, mean QF_ICRP_ and mean RBE_γacute_ for the different shielding materials are summarized in Table 2.

**Table 2:** Variation of the relative effective dose equivalent, H_E_, with shielding materials of differing mass thickness. The relative neutron contribution rates (H_E(ICRP)neutron_) and contributions to the charged particle contributions (H_E(ICRP)_) are given.

Shielding material	Mass thickness (g/cm^2^)	Relative absorbed dose	Mean QF_ICRP_	Relative H_E(ICRP)_	Relative H_E(ICRP)neutron_	Mean RBE_γacute_	Relative H_E(RBEγacute)_
	0	1.00	3.23	1.00	0	1.48	1.00
Al	5	0.91	2.88	0.81	0.01 (1.2%)	1.41	0.87
	10	0.87	2.61	0.70	0.02 (2.6%)	1.35	0.79
	15	0.83	2.41	0.62	0.03 (4.1%)	1.30	0.73
	20	0.79	2.25	0.55	0.03 (5.9%)	1.26	0.68
PE	5	0.90	2.67	0.75	0.01 (1.2%)	1.36	0.83
	10	0.85	2.32	0.61	0.02 (2.6%)	1.28	0.73
	15	0.79	2.09	0.51	0.02 (4.2%)	1.22	0.66
	20	0.75	1.94	0.45	0.03 (5.7%)	1.18	0.60
CFRP	5	0.90	2.76	0.77	0.01 (1.2%)	1.38	0.84
	10	0.85	2.44	0.64	0.02 (2.6%)	1.31	0.75
	15	0.80	2.21	0.55	0.02 (4.2%)	1.25	0.68
	20	0.76	2.04	0.48	0.03 (5.9%)	1.21	0.62

## Discussion

The calculated total absorbed dose and H_E(ICRP)_ in this study were 10–15% and ~ 20% lower than previous interplanetary measurements, respectively^6,39^. Here, we note that H_E(ICRP)_ was compared with the measured dose equivalents. The effective dose equivalent, which is a protection value, is determined by normalizing dose equivalent of human body, which is an operational value, with tissue or organ weighting factors. Although the effective dose equivalent is not equal to the dose equivalent, their comparison is reasonable considering the differences in irradiation targets (human body for our calculations and detector mediums for measurements). Considering discrepancies between the simple calculation geometry and actual measurement configuration, measurement uncertainties, the differences in the solar modulation factor, and contribution from secondary particles, which were not included in our calculation, these results are almost consistent. The reduction rates of H_E(ICRP)_ by material shielding in this study were 45–55% at 20 g/cm^2^, which are also similar values to previous calculations considering the above discrepancies^38,52^. There was an important difference between RBE_γacute_ and QF_ICRP_ in the ratio of light and heavy ions (e.g., H vs. Fe): the QF_ICRP_ value of Fe was 11–12 times higher than that of H, compared to 3.5 times higher for RBE_γacute_. This difference implies that the contribution of heavy ions to H_E(ICRP)_ is ~ 3 times higher than that to H_E(RBEγacute)_.

Comparing Figs. 3 and 5 indicates that the low energy edge of H does not contribute to the H_E_ spectra and a small enhancement due to Mg and Si makes a contribution to LET of ~ 30 keV/µm. Because the kinetic energy of the secondary H around the edge is ~ 1 MeV, its energy deposition is not large enough to increase the dose. Meanwhile, heavy ions, such as C and O, for LET ~ 10 keV/µm are relativistic, which increases the dose. The penetrative ability of ions is an important factor when considering the energy deposition in the human body. Therefore, the underestimation of ions with a lower energy of < 1 MeV/n was not significant. Like the H edge, the shielding materials diminished the He edge at 20–100 keV/µm, inducing a small enhancement in Mg and Si.

The reduction in the absorbed dose mainly came from the fragmentation of HZE particles. Around the mean energy of the GCR particles (~ 1 GeV/n), the energy loss in the shielding materials, which depended on the stopping power, was not significant. The mean QF_ICRP_ and RBE_γacute_ values decreased as a function of shielding thickness. The reduction rate of HZE particles by fragmentation in the shielding materials was larger than the rate of increase in protons. At a typical mass thickness of 20 g/cm^2^, the mean QF_ICRP_ and RBE_γacute_ decreased to 30% and 15% for Al, 40% and 20% for PE, and 37% and 18% for CFRP, respectively. The reduction rates of mean RBE_γacute_ were lower than those of mean QF_ICRP_ because the heavy ion contributions to mean QF_ICRP_ were larger than those to mean RBE_γacute_. The relative variations in H_E_ values among the shielding materials were obtained from the relative absorbed doses and relative mean QF_ICRP_ or RBE_γacute_ (Eq. 3). At a thickness of 20 g/cm^2^, the reduction rates of H_E(ICRP)_ and H_E(RBEγacute)_ were 45% and 32% for Al, 55% and 40% for PE, and 52% and 38% for CFRP, respectively. PE achieved ~ 24% and ~ 5% higher reduction rates than Al and CFRP, respectively. An idea for deep space missions is to construct some spacecraft parts made of CFRP, which has a better shielding capability than conventional Al based materials. Our previous evaluation implied that the material switching from Al to CFRP at the same actual thickness provided a similar dose reduction despite that the total mass of CFRP module was much smaller than that of Al by a factor of their density ratio^41^. The actual shielding materials are not only spacecraft materials but also fuel, water and other supplies. The materials to construct a spacecraft will be selected by not only radiation shielding performance but also many requirements such as thermal property, ultraviolet resistance, moisture absorption resistance and so on. If the complete material switching from Al to CFRP is attained by the same mass thickness (g/cm^2^), CFRP will give a benefit for the protection of crews from GCRs.

The results were discussed for charged particles from protons to Fe ions as mentioned in the results section; the contributions of neutrons, photons, pions, and muons were not considered. In particular, the neutron contribution to the dose equivalents may not be small among secondary radiation particles (e.g., Refs.^4,5^). The neutron contributions obtained from the ICRP conversion coefficients, H_E(ICRP)neutron_, are also listed in Table 2. The neutron contribution rate was much lower than the charged particle contributions (5.9% of the H_E(ICRP)_ at most). The fragmentation reactions of the primary particles, which produce neutrons, occur effectively in light shielding materials. The higher neutron contribution in Al indicates a high thermalization with hydrogen atoms in PE and CFRP. RBE_γacute_ for neutrons is one of issues to be addressed in future.

**Figure 6:**
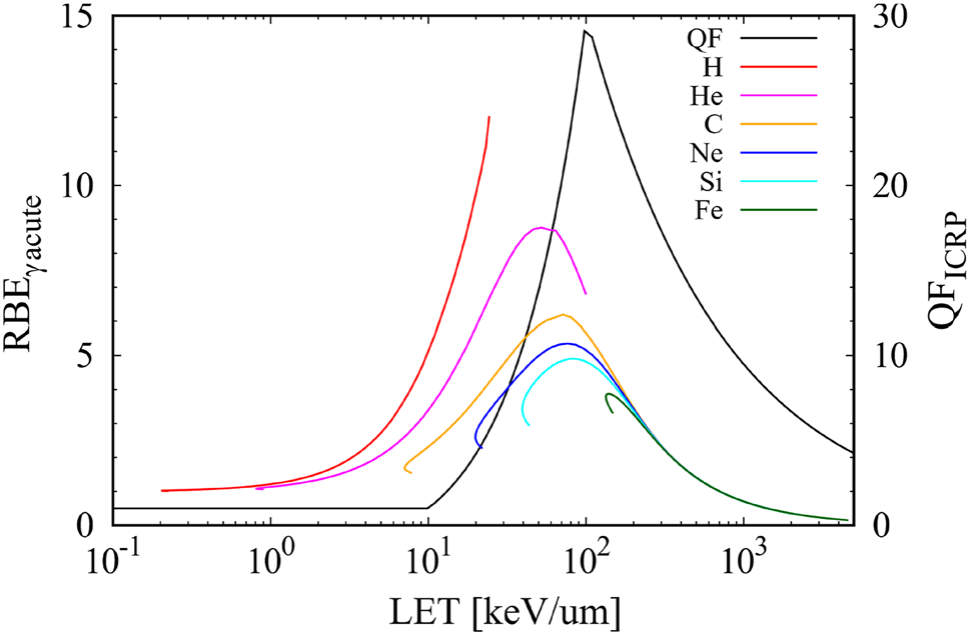
LET dependencies of biological scale parameters. RBE_γacute_ (left axis) for simple exchanges to human peripheral blood lymphocytes^22^ and QF_ICRP_ (right axis)^15^.

The difference between QF_ICRP_ and RBE_γacute_, except for the reference gamma-ray, is in the models: QF_ICRP_ depends on the energy deposition (i.e., LET) while RBE_γacute_ depends on the charge and energy of the incident particles. The energy of the secondary particles is dependent on that of the primary particles. One possible explanation for the larger QF_ICRP_ peak than RBE_γacute_ peaks in their LET dependencies (Fig. 6) is the difference in targeted radiation for dose assessment. Heavy ions at their peaks have relativistic energy, which produces relativistic secondary particles. The fact that the RBE_γacute_ peaks are lower than the QF_ICRP_ peak reflects the significant contribution of low energy secondary particles because electrons are biologically effective at low energies (< 10 keV)^10^.

The advantages and disadvantages of QF_ICRP_ and RBE_γacute_ are summarized in Table 3. The RBE_γacute_ evaluates relative biological effectiveness with a smaller uncertainty than RBE_max_ relevant to QF_ICRP_, as mentioned in the introduction. One of the difficulties in using RBE_γacute_ is the selection of the parameters. The selected parameters, and thus RBE_γacute_, depend on the targeted radiation field and biological effects. Thus, the obtained dose equivalent was not comparable in different radiation environments. Meanwhile, QF_ICRP_, which is determined by LET, offers good usability. However, the effective dose equivalents and QF_ICRP_ have been established for general dose assessment on the ground and have been replaced with the effective dose^15^. The radiation environment on the ground is primarily composed of photons from natural radioisotopes. Other contributors to this environment are protons, alpha particles, and neutrons in specific radiation fields, such as medical accelerators and nuclear power plants. HZE particles were not the main target of QF_ICRP_ on the ground. QF_ICRP_ has not been updated since the 1990 ICRP recommendation^15^. The establishment of a standard parameter specified for a mixed radiation environment occupied by protons and heavy ions is necessary for practical dose assessment in deep space.

**Table 3:** Advantages and disadvantages for QF_ICRP_ and RBE_γacute_.

	Advantages	Disadvantages
QF_ICRP_	Uniquely determined by LET Traditionally used and easy to compare in different radiation environments	Targeted for exposure on the ground Not updated since 1990
RBE_γacute_	Smaller uncertainty than RBE_max_ (QF_ICRP_) Based on measured data of HZE particles	Parameters depend on targeted radiation field and biological effects Difficult to compare in different radiation environments

## Conclusion

We investigated the radiation shielding performance and effective dose equivalents based on the ICRP radiation QF and the plausible RBE for three shielding materials, Al, PE, and CFRP, using the Geant4 Monte Carlo simulation. The QF_ICRP_ values of Fe were 11–12 times larger than those of H, compared to ~ 3.5 times larger for RBE_γacute_. Therefore, the contribution of heavy ions to H_E(ICRP)_ was larger by a factor of ~ 3 compared to that to H_E(RBEγacute)_. The shielding materials reduced the flux of primary particles heavier than He due to projectile fragmentation. The flux reduction rate increased with successively heavier nuclei and the increase was particularly large for heavier ions. The shielding materials efficiently reduced the effective dose equivalent due to ions with QF_ICRP_ > 3.36 and RBE_γacute_ > 2.26. The reduction rates of H_E(ICRP)_ were higher than those of H_E(RBEγacute)_ because of the large contribution of heavy ions. The expected radiation exposure risk was reduced by 50% for QF_ICRP_ and 38% for RBE_γacute_ when using 20 g/cm^2^ CFRP. Therefore, a spacecraft made of CFRP could improve the radiation shielding performance compared to conventional Al-based spacecraft and help mitigate space radiation hazards in future space missions. The discrepancy between QF_ICRP_ and RBE_γacute_ highlighted the necessity of a new standard for mixed radiation environments occupied with protons and heavy ions in deep space.

## Methods

We calculated the fluences of GCR protons and heavy ions up to Fe (Z = 1–26) with energies ranging from 1 MeV/n to 100 GeV/n in free space and with shielding materials using Monte Carlo simulation with Geant4 ver. 10.04.02^53–55^. The primary particles in the energy region of 1–10 MeV/n (Fig. 1 dotted lines) did not contribute to the flux behind the materials because these low energy particles were stopped within a thin shielding layer of only a few micrometers. Primary particles in this energy range were excluded from the projectile to reduce the calculation cost. The mean QF_ICRP_ and RBE_γacute_ values were obtained, including the particles in this energy range. Al, PE, and CFRP with mass thicknesses of 5, 10, 15, and 20 g/cm^2^ were employed as shielding materials. The GCR source was derived from the DLR model during the solar minimum phase^56^. The solar minimum assumption provides the worst case for radiation exposure. The number of each primary ion was fixed at 10^6^ to obtain the total dose rate by merging all the dose rates. The elemental composition of the CFRP was assumed to be that of a commercial composite material, CF/PEEK (Toray Cetex TC1200, Toray Advanced Composites, USA). The dose and LET changes due to nuclear fragmentation simulated by Geant4 have been experimentally validated in our previous studies^40,41^.

The absorbed dose (D) for the whole human body, due to each particle in free space and behind the target materials, was obtained from the particle fluences and ICRP conversion coefficients for isotropic exposure^9^. The ICRP defines the QF_ICRP_ as the following^15^:

2 $$Q{F}_{ICRP}=\left\{\begin{array}{c}1\\ 0.32 LET-2.2\\ 300/\sqrt{LET}\end{array}\right. \begin{array}{c}(LET<10)\\ (10<LET<100)\\ \left(LET>100\right).\end{array}$$

The mean RBE_γacute_ was obtained through the charge and energy dependences for the targeted effects model as follows^21,22^:

3 $$\begin{array}{c}RB{E}_{\gamma acute}=(1-P)+6.24{\sigma }_{0}/{\alpha }_{\gamma }LET\\ P={(1-exp(-{Z}^{*2}/\kappa {\beta }^{2}))}^{m},\end{array}.$$

where ${Z}^{*}$ and $\beta$ are the effective charge number of the particle and particle velocity relative to light, respectively; parameters ${\sigma }_{0}$, *m*, and $\kappa$ are constants based on radiobiological experiments; and ${\alpha }_{\gamma }$ is the linear regression coefficient for the acute dose of gamma-rays at the same endpoint. We employed these parameters for simple exchanges with human peripheral blood lymphocytes from Cacao et al.^22^ since blood is uniformly distributed in the whole body. Here, the dose rate in space is of the order of hundreds of micro grays per day e.g., Refs.^6,33,34,37,43,57^. Non-targeted effects are expected to be negligible at sufficiently low doses of < 1 mGy e.g., Refs.^58–61^ and a large number of DNA double-strand breaks are required for complex exchanges. Therefore, we selected a targeted effects model and assumed a simple exchange with human lymphocytes. Although the parameters studied in previous works^17,21^ provided 2–5 times larger RBE_γacute_ values, these studies targeted solid cancer or leukemia, which are also relevant to nontargeted effects and complex exchanges. Other parameters must be used to target these critical radiation hazards. The RBE_γacute_ and QF_ICRP_ values of major GCR particles as a function of LET are presented in Fig. 6^15,22^. The LET range of each particle corresponded to an energy range of 1 MeV/n–10 GeV/n. The dose equivalent H_E_ was obtained by the products of the absorbed dose and the QF_ICRP_ or RBE_γacute_:

4 $${H}_{E}= \Sigma {D}_{i} Q{F}_{ICRP i} \left(or RB{E}_{\gamma acute i}\right),$$

where $i$ indicates the GCR particle (Z = 1–26).

## Data Availability

The datasets generated in this study are available from the corresponding author upon reasonable request. There were no restrictions in terms of data availability.
